# Disease Course of Korean African Swine Fever Virus in Domestic Pigs Exposed Intraorally, Intranasally, Intramuscularly, and by Direct Contact with Infected Pigs

**DOI:** 10.3390/v16030433

**Published:** 2024-03-11

**Authors:** Ki-Hyun Cho, Seong-Keun Hong, Da-Young Kim, Hyun-Joo Sohn, Dae-Sung Yoo, Hae-Eun Kang, Yeon-Hee Kim

**Affiliations:** 1 Foreign Animal Disease Division, Animal and Plant Quarantine Agency, 177 Hyeoksin 8-ro, Gimcheon 39660, Republic of Korea; vet10@korea.kr (K.-H.C.); hongsky@korea.kr (S.-K.H.); kdy04207@naver.com (D.-Y.K.); shonhj@korea.kr (H.-J.S.); kanghe@korea.kr (H.-E.K.); 2 College of Veterinary Medicine, Chonnam National University, Gwangju 61168, Republic of Korea; shanuar@jnu.ac.kr

**Keywords:** African swine fever, animal experiment, inoculation routes, disease course, comparison

## Abstract

African swine fever (ASF) is a fatal contagious disease affecting swine. The first Korean ASF virus (ASFV) isolate (Korea/Pig/Paju1/2019) was used to compare the disease course of ASFV in pigs inoculated via the four routes. In the challenge experiment, domestic pigs were infected via the intraoral (IO) and intranasal (IN) routes with a 10^6^ 50% hemadsorbing dose (HAD_50_) and an intramuscular (IM) injection of 10^3^ HAD_50_. In the direct contact (DC) group, five naïve pigs were brought into direct contact with two IM-ASFV-infected pigs. IO-, IN-, and IM-inoculated pigs showed similar disease courses, whereas DC pigs had comparable ASF syndrome after a 7-day latent period. The disease course in the DC route, one of the most common routes of infection, was not significantly different from that in the IO and IN routes. IM and DC groups differed in terms of the severity of fever and hemorrhagic lesions in the lymph nodes and spleen, indicating that the IM route, suitable for early vaccine development trials, is not appropriate for studying the ASFV infection mechanism, including early stage of infection, and IO and IN challenges with a designated dose can be alternatives in trials for assessing ASFV pathogenicity and vaccine efficacy investigations.

## 1. Introduction

African swine fever (ASF) is a contagious disease that affects domestic pigs and wild suids. ASF, which is characterized by acute hemorrhagic fever and subsequent death, with mortality reaching 100%, causes enormous socioeconomic losses in affected countries. ASF was first described in Kenya in 1921 and confined to sub-Saharan African countries. Two epidemics of ASF out of Africa have occurred. The first epidemic began in 1957 and was terminated in 1999 in Europe, except for in Sardinia, Italy [1]. When ASFV suddenly emerged at a pig farm in Georgia in 2007 [2], the second epidemic started and unprecedented pandemics have continued [3]. ASF rapidly transmitted from Georgia to the other Caucasus countries and Russia and has expanded westward and eastward [4]. From 2013, 22 European countries—Georgia, Armenia, Russia, Azerbaijan, Ukraine, Belarus, Lithuania, Latvia, Estonia, Poland, Moldova, Bulgaria, Hungary, Romania, Belgium, Slovakia, Servia, Greece, Germany, Italy, Bosnia-Herzegovina, and Sweden—were affected [5]. The ASF situation in Asia has become more aggravated than that in Europe. The first ASF outbreak in Asia was reported in China in August 2018 [6], which caused extensive economic losses [7]. Since then, the spread of ASF has accelerated from there. As of December 2023, 18 Asian countries—China, Mongolia, Vietnam, Cambodia, North Korea, Laos, Myanmar, the Philippines, South Korea, Timor-Leste, Indonesia, India, Malaysia, Bhutan, Thailand, Nepal, Singapore, and Bangladesh—have reported ASF cases. In meanwhile, ASF has spread to Oceania (Papua New Guinea) and America (Dominican Republic and Haiti) [5]. ASF is still widespread in the affected continents.

The ASF virus (ASFV) is the etiological agent of ASF. ASFV is the only member of the genus *Asfivirus*, family *Asfarviridae,* and is a large enveloped DNA virus [8]. The viral genome is 170–194 kbp in length and encodes >60 structural proteins and 100 non-structural proteins [9]. ASFV strains are divided into 24 genotypes based on analysis of partial p72 sequences [10]. Twenty-four genotypes of ASFV strains have been detected in Africa [11]. Only genotype I virus circulated during the first epidemic (1957–1999) in Europe and America, and it has persisted in Sardinia, Italy [12]. The second epidemic (2007 to date) has continued with genotype II ASFV [2], but several genotype I viruses were confirmed in China [13,14]. Recently, a new analysis of full-length p72 protein sequences revealed that 24 genotypes can be reduced to 6 genotypes [15]. However, high genetic diversity at the whole viral genome level exists, which can be an implication for the efficacy of live attenuated vaccines [16]. ASFV strains are classified into highly, moderately, and low-pathogenic strains, which induce four clinical courses in pigs, peracute, acute, subacute, and chronic forms [17]. Although most ASFV strains circulating in Asia and Europe were highly pathogenic [18,19,20,21,22,23], attenuated viruses were increasingly being detected in Eurasia [13,14,24,25].

ASF transmission occurs through direct contact between infected and susceptible pigs; however, fomites, e.g., contaminated feed, water, equipment, and clothing, can also spread the disease [17]. Under natural conditions, the principal transmission route in domestic pigs is direct contact with the excreted virus through nuzzling and/or ingestion [26]. Arthropod transmission via several *Ornithodoros* ticks is another possible transmission from wild suids to domestic pigs and within domestic pig populations. However, because competent *Ornithodoros* ticks are found only in Africa, North America, and the Iberian Peninsula of Europe [27], the possibility of ASFV transmission via soft ticks is low in Asian countries, including South Korea. Owing to the highly reproducible clinical course of ASFV, an extensive portion of animal experiments in the research area of ASF, including the estimation of pathogenicity and ASFV vaccine assessment, have been conducted using intramuscular (IM) inoculation. However, this route bypasses many factors involved in the innate immune defense mechanism on the oral and upper respiratory mucosal surfaces that the virus normally encounters during natural infection. Therefore, the IM challenge, an unnatural method, creates limitations in understanding the clinical course of natural infections.

In South Korea, the first ASF outbreak that occurred at a pig farm in Paju City, located in the northwestern border region of North Korea, was detected on 16 September 2019 [28]. From 2 October 2019 to December 2023, 38 domestic pig farms and 3490 wild boars were confirmed in the country [29]. The first Korean ASFV strain, designated as Korea/Pig/Paju1/2019, was isolated from the spleen of an ASFV-positive dead pig under the index premise. Genetic analysis of Korea/Pig/Paju1/2019 indicated that it belonged to the p72 genotype II and CD2v serogroup 8, with a central variable region 1 and an intergenic region between I73R and I329L II variants [28]. The whole genome of Korea/Pig/Paju1/2019 was 99.98% identical to that of Georgia 2007/1. The Korean strain was shown to be highly virulent, causing peracute and acute forms of the disease in an IM challenge experiment [22]. Information on the disease progression of Korean ASFV strains by infection route is unavailable elsewhere. In this study, animal experiments were performed to define the clinical signs, viremia, viral shedding, and post-mortem lesions in peracute or acute forms of ASF induced by intraoral (IO), intranasal (IN), IM, and direct contact (DC) routes.

## 2. Materials and Methods

### 2.1. Virus

The first Korean ASFV isolate, designated Korea/Pig/Paju1/2019, was used. The ASFV strain was isolated from the spleen (cycle threshold [Ct] = 17.1) of a dead ASFV-positive pig on 16 September 2019. The inoculum was prepared after two passages of porcine pulmonary alveolar macrophages (PAMs). Titrations of the inoculum were estimated by a hemadsorption assay to monitor the endpoint of dilution of the strain in PAMs, according to the manual of the European Union Reference Laboratory of ASF [30].

### 2.2. Animal Experiment Designs

Twenty-four 8-week-old Landrace pigs from a commercial farrow-to-finish pig farm were used for the animal experiments. They were free of swine infectious diseases, including ASF, classical swine fever, porcine respiratory and reproductive syndrome, porcine epidemic diarrhea, porcine transmissible gastroenteritis, porcine parvovirus, Aujeszky’s disease, and foot-and-mouth disease. All the pigs tested negative for ASFV antigens and antibodies. All pigs were introduced into the ABSL-3 facility, randomly grouped, and allowed to acclimatize to the new environment for five days. All the animals were fed daily and provided access to water ad libitum.

Experiments were performed to compare the three routes of direct inoculation, namely the IO, IN, and IM routes, and DC. Five pigs were assigned to the IN, IM, and DC groups, respectively, and four pigs were assigned to the IO group. The most commonly used dose for IM challenge in assessing the pathogenicity and immunoprotective effect of the ASFV vaccine is 10^2^ or 10^3^ HAD_50_ [21,22,23,31,32,33,34]. IO and IN inoculations required approximately 10^2^–10^4^ times the viral titer in the inoculum to induce similar disease courses in pigs injected intramuscularly in an animal experiment with a highly pathogenic strain [35]. Taken together, inoculation titers by IO, IN, and IM routes were determined. In the IO and IN groups, assigned pigs were sedated and anesthetized with intramuscular administration of Zoletil^®^ (tiletamine hydrochloride and zolazepam hydrochloride; Virbac Laboratories, Carros, France) and Rompun^®^ (xylazine hydrochloride; Bayer, Leverkusen, Germany). After placing the pigs in sternal recumbency, pigs in the IO and IN groups slowly received 1 mL of the inoculum, a 10^6^ 50% hemadsorbing dose (HAD_50_), via a 1 mL syringe into the mouth or nostril, respectively, and were constrained in the same position for at least 5 min to ensure infection. In the IM group, five pigs were challenged with 1 mL of the inoculum at 10^3^ HAD_50_ within the semimembranosus muscle, of which two IM-ASFV-infected pigs were co-housed with the DC group. The control group consisted of five pigs (mock-infected group).

### 2.3. Clinical Observation and Sample Collections

All pigs were monitored for clinical signs from the day of inoculation until death or euthanasia. The clinical signs in each pig were observed and recorded daily according to the method previously described [36]. The clinical scoring system comprised 10 categories: fever, loss of appetite, recumbency, skin hemorrhage, joint swelling, respiratory difficulties, ocular discharge, diarrhea/bloody diarrhea, hematuria, and vomiting. The clinical scores ranged from 0 to 40. The humane endpoint for euthanasia was determined as described in a previous study [24]. The pigs subjected to euthanasia were deeply anesthetized with intramuscular injection of Zoletil^®^ and Rompun^®^ and exsanguinated. Necropsy was performed on all dead and euthanized pigs. When all the pigs in the IO, IN, IM, and DC groups had died, the pigs in the control group were euthanized after deep anesthesia with IM injection of Zoletil^®^ and Rompun^®^ and exsanguination.

Whole blood samples with ethylenediamine tetraacetic acid (EDTA), serum, and oral, nasal, and rectal swab samples were collected daily from the day of inoculation to the day before death or euthanasia. After manually restraining the pigs, the whole blood and serum were collected from the external jugular vein. Sterile cotton swabs in a transport medium (Noble Biosciences, Hwaseong, South Korea) were used for swab sampling. Tissue samples from the spleen, liver, kidneys, heart, forelimb muscles, and five lymph nodes (mandibular, inguinal, mesenteric, gastrohepatic, and renal lymph nodes) were collected to assess the ASFV loads.

### 2.4. Laboratory Test for ASFV Antigen and Antibody Detection

Viral nucleic acids were extracted from all collected samples using the Maxwell^®^ RSC 48 instrument (Promega, Madison, WI, USA) according to the manufacturer’s instructions. The Maxwell^®^ RSC Whole Blood DNA kit and Maxwell^®^ RSC Viral Total Nucleic Acid purification kits were used for whole blood with EDTA and other samples including tissues and swabs. World Organization for Animal Health (WOAH) TaqMan^®^ quantitative polymerase chain reaction was conducted using the BioRad CFX-96 detection system [37]. Samples with a recorded Ct of <40.0 were considered positive, whereas samples without recorded Ct values were considered negative. Viral genome copy numbers were estimated based on the standard curve between the Ct value by WOAH TaqMan^®^ qPCR and copy numbers of pUC57 vectors inserted with partial B646L genes of BA71V strain [22]. Sera were screened for the presence of ASFV antibodies using the INgezim PPA COMPAC K3 kit (Gold Standard Diagnostics, Madrid, Spain) according to the manufacturer’s instructions. To confirm samples with positive and inconclusive enzyme-linked immunosorbent assay results, the immuno-peroxidase test was performed following standard protocols provided by the European Reference Laboratory for ASF [30].

### 2.5. Statistical Analyses

The Kruskal–Wallis test by ranks, a non-parametric alternative to one-way analysis of variance, was performed to identify any differences in total survival days, onset of clinical signs, maximum clinical score, onset of viremia, and virus shedding via oral, nasal, and rectal routes between the experimental groups, considering that these variables did not follow normality and homoscedasticity assumptions. For the DC group, a latent period of 7 days was deduced from the analyzed parameters. Additionally, the Bayes factor was estimated to identify the differences in the distribution of the maximal change in temperature and clinical scores from day 0 between the control group and each experimental group. Statistical analyses were conducted using R software (version 4.3.1; https://r-project.org, accessed on 24 July 2023).

## 3. Results

### 3.1. Disease Progression and Clinical Signs

In the IO, IN, and IM groups, fever (>40 °C) was first detected at 4.5 ± 0.6 (4–5), 4.2 ± 0.5 (4–5), and 4.6 ± 0.9 (4–6) days post-inoculation (dpi), respectively (Table 1). The fever continued until the day before death or euthanasia (4–10 dpi) of all pigs, except for in pig number (no.) 1, in which the rectal temperature decreased to normal (38.8 °C) on the day before death or euthanasia (9 dpi). In three groups, fever, inappetence, depression, and recumbency were commonly observed. However, fever was more severe (>41 °C) in the IO and IN groups than in the IM group. Some pigs exhibited skin hemorrhage (2/4 in the IO group and 2/5 in the IM group), ocular discharge (2/5 in the IN group), joint swelling and lameness (2/5 in the IN group), difficulty in breathing (1/5 in the IM group), and diarrhea (1/5 in the IM group). Pig nos. 7 and 13 had fever at 4 dpi and died without any significant clinical signs at 5 and 6 dpi, respectively. The clinical scores in all pigs excluding pig nos. 7 and 12 increased to >12 (12–18) points on the day before death or euthanasia (4–10 dpi). Pigs in the IO group died or were euthanized at 8.8 ± 1.0 (8–10) dpi. Survival periods of IN and IM groups were 8.6 ± 2.3 (5–11) and 8.0 ± 1.2 (6–9) dpi, respectively (Table 1). The DC group began to have fever (>40 °C) at 11.6 ± 0.9 (11–13) dpi. Clinical signs were first observed at 13.2 ± 0.5 (13–14) dpi. Severe fever (>41 °C), loss of appetite, depression, recumbency, and difficulty breathing were observed in all pigs. Some pigs in this group presented with skin hemorrhage (pig nos. 15 and 17) and diarrhea (pig nos. 16 and 17). The clinical score increased by >14 points. Pigs in the DC group died or reached the humane end point at 16.6 ± 1.5 (15–18) dpi. In the control group, rectal temperature was normal (<40 °C) and no clinical signs were observed.

### 3.2. Viremia, Virus Shedding, and Antibody

**Figure 1:**
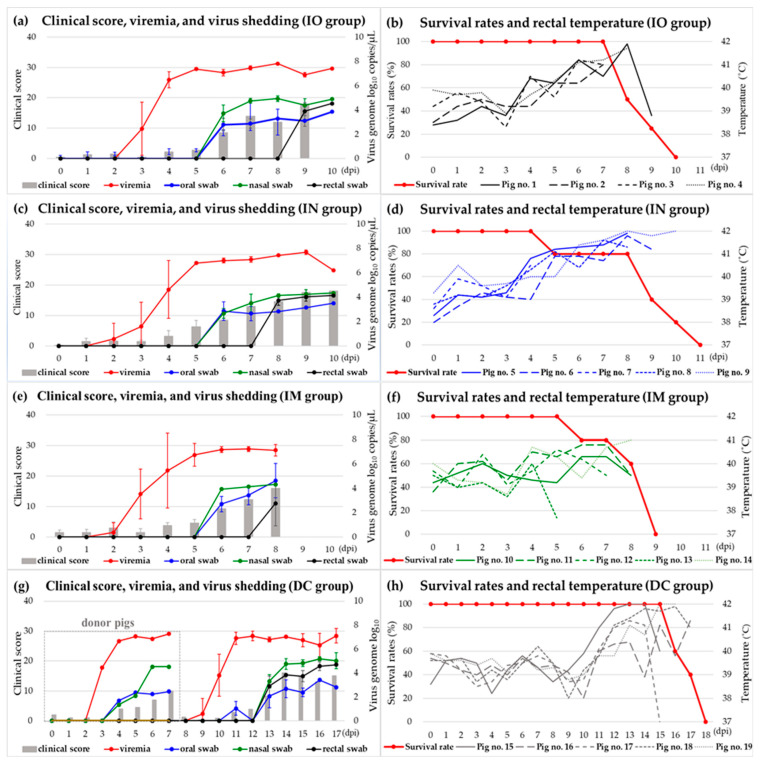
Results of evolution of the clinical score, viremia, and virus shedding via the oral, nasal, and rectal routes (**a**,**c**,**e**,**g**) and rectal temperature and survival rates (**b**,**d**,**f**,**h**) in the IO, IN, IM, and DC groups. IO, intraoral; IN, intranasal; IM, intramuscular; DC, direct contact; no., number.

In the IO group, onset of viremia was detected at 3.3 ± 0.3 (3–4) dpi. The viral load was 10^2^–10^5^ genome copies/µL on the day of the first detection (3–4 dpi). Its level increased to >10^6^ genome copies/µL and continued until the day before death or euthanasia (7–9 dpi). Virus genome was detected in oral and nasal swabs at 4.8 ± 0.3 (4–6) and 4.3 ± 0.1 (4–5) dpi, respectively. Virus loads in both swab samples were 10^1^–10^2^ genome copies/µL at the first detection (4–6 dpi) and increased to 10^3^–10^5^ genome copies/µL. The ASFV genome was detected at 5.5 ± 2.3 (4–9) dpi in rectal swabs. Virus loads started at 10^1^–10^4^ genome copies/µL. Virus shedding via the oral and nasal routes continued before death or euthanasia (7–9 dpi). By contrast, the viral genome was intermittently detected in rectal swabs collected from some pigs (pig nos. 2 and 3). In the IN group, viremia began at 3.2 ± 0.4 (2–4) dpi, followed by oral and nasal shedding at 4.4 ± 0.6 (4–5) and 5.0 ± 0.9 (4–6) dpi, respectively. Viral load in the blood was 10^1^–10^5^ genome copies/µL initially, increased to 10^6^–10^7^ genome copies/µL within 1 day after the first detection (2–4 dpi), and was maintained until the day before death or euthanasia (4–10 dpi). The viral load in oral and nasal swabs was first detected at 10^1^–10^2^ genome copies/µL (4–6 dpi). Their loads increased to 10^3^–10^4^ genome copies/µL. Rectal shedding began at 5.0 ± 0.9 (6–8) dpi with 10^2^–10^3^ genome copies/µL, which increased to 10^2^–10^3^ genome copies/µL. In pig no. 7, the ASFV genome was not detected in the rectal swabs. Similar viremia and viral shedding patterns were observed in the IM group (Figure 1). In pig no. 13, no ASFV genome in rectal swabs was detected at any time point in the experiment.

In the DC group, viremia was first detected at 9.8 ± 0.1 (9–10) dpi and continued until death. ASFV loads were 10^1^–10^4^ genome copies/µL at the first detection (9–10 dpi), and they increased to 10^6^–10^7^ genome copies/µL 1 day later and were maintained until the day before death or euthanasia (14–10 dpi). Oral and nasal shedding occurred at 7.0 ± 1.2 (6–8) and 6.0 dpi, respectively, and was intermittently detected. The viral load was 10^1^–10^2^ genome copies/µL. In both swab samples, the ASFV genome was continuously detected from 11 dpi at 10^1^–10^3^ genome copies/µL and 11.6 ± 0.6 (11–12) dpi to death at 10^3^–10^5^ genome copies/µL (Figure 1a–h). In the control group, the ASFV genome was not detected in any blood and swab samples.

The IO, IN, IM, and DC groups showed no statistically significant differences in most of the analyzed transmission parameters, except for the onset of clinical signs (*p* = 0.043) and maximum titers of nasal shedding (*p* = 0.046) (Table 1). Compared with the control group, the IO (Bayes factor; BF = 14509.7), DC (BF = 1116.0), IM (BF = 32.4), and IN (BF = 30.6) groups showed different maximum clinical scores with decisive or very strong evidence. Rectal temperature showed statistically significant differences with substantial, very strong, and strong evidence in the IN (BF = 51.7), DC (BF = 11.6), and IO (BF = 3.4) groups, respectively. However, no statistically significant differences were observed between the control and IM groups (BF = 0.4).

### 3.3. Gross Lesions at Necropsy and Viral Loads by Organ

**Figure 2:**
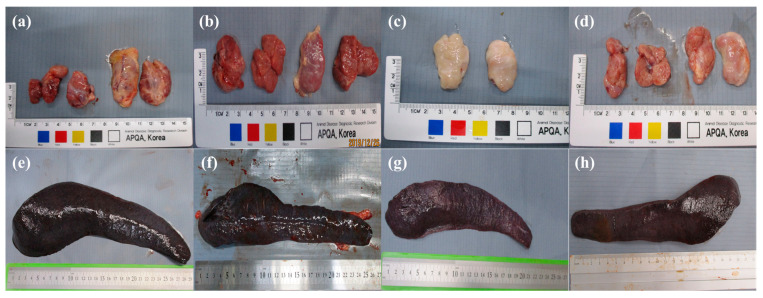
Gross lesions of spleen and lymph nodes. (**a**,**b**) Enlargement and hemorrhage in inguinal lymph nodes (pig nos. 4 and 19); (**c**) enlarged inguinal lymph node (pig no. 5); (**d**) enlarged and slightly hemorrhagic mandibular and inguinal lymph nodes (pig no. 14); (**e**,**f**) severely thickened, enlarged, dark, and friable spleen (pig nos. 1 and 18); (**g**) dark spleen (pig no. 5); and (**h**) spleen without any pathological lesions (pig no. 12).

Pathological lesions observed in all groups were enlarged lymph nodes. Hemorrhage in five lymph nodes was observed in all pigs in the IO and DC groups. Some pigs in the IN and DC groups did not have hemorrhages in the mandibular, inguinal, or mesenteric lymph nodes. Petechiae in the kidneys (17/19) were observed in all pigs, except for one pig in each of the IN and IM groups, respectively. All pigs in the IO group had thickened, enlarged, dark, and friable spleens. Pathological lesions of the spleens in the IN and DC groups were slight, and the IM group showed less severe gross lesions in the spleen than the other groups. Hydropericardium (16/19) and interstitial pneumonia (14/19) were observed in most pigs. The control group did not show any gross lesions at necropsy. The pathological lesions are summarized in Table 2 and gross lesions in the spleen and lymph nodes are shown in Figure 2.

The ASFV genome was detected in all the organs obtained from dead or euthanized pigs in the IO, IN, and DC groups. Commonly, the largest amount of ASFV genomes (10^5.9^–10^6.4^ genome copies/µL) was detected in the spleen of all groups, followed by in the liver (10^5.1^–10^5.9^ genome copies/µL), mandibular lymph nodes (10^4.8^–10^5.8^ genome copies/µL), kidney (10^4.3^–10^4.9^ genome copies/µL), heart (10^4.1^–10^4.8^ genome copies/µL), and forelimb muscle (10^3.4^–10^3.8^ genome copies/µL). In the control group, all obtained organs were ASFV-negative.

## 4. Discussion

In this study, we conducted animal experiments to compare the disease course of ASF induced in domestic pigs via the IO, IN, IM, and DC routes. Pigs in the IM group inoculated with 10^3^ HAD_50_ died within 8.0 ± 1.2 dpi, whereas those in the IO and IN groups challenged with 10^6^ HAD_50_ died or reached the humane end point within 8.8 ± 1.0 and 8.6 ± 2.3 dpi, respectively. The latent period of the IM group was 4.4 ± 0.9 days, similar to those of the IO (4.0 ± 0 days) and IN groups (4.4 ± 0.5 days). All pigs in the three groups had peracute or acute ASF. The clinical scores of the IO, IN, and IM groups differed significantly from that of the control group; however, changes in rectal temperature in the IM group did not show any statistically significant differences. After 7 days of the latent period, the DC group showed an acute disease course similar to that in the IO, IN, and IM groups. The four groups showed statistical differences in the onset of clinical signs (*p* = 0.043) and maximum titers in nasal swabs (*p* = 0.046) among the four groups. Pigs in the IO, IN, and DC groups, except for pig no. 7, had a higher fever (>41 °C), even reaching 42 °C, and tended to have more congested and hemorrhagic lesions in the spleen and lymph nodes than pigs in the IM group. These results correspond to the fact that distinct hemorrhagic lesions were observed in the spleen and lymph nodes of an ASFV-positive pig with high fever in the index farm of South Korea [28]. DC transmission is one of the most common routes of infection. In this experiment, IO and IN inoculations with a designated infection dose stimulated natural infections, similar to the DC route. IO and IN routes can be used for elucidating the natural infection mechanism with ethical reason and convenience in place of the DC route.

Studies have been conducted to determine the 50% pig infectious dose (PID_50_), i.e., the minimum dose required to infect 50% of pigs. A total of 10^5^ HAD_50_ was necessary to infect pigs via the IO route, according to Maurer and Griesemer [38]. A low ASFV dose (<10^5^ TCID_50_) for IM and a high dose (>10^3^ TCID_50_) for IO have been reported to establish ASFV infection in domestic pigs [39,40,41,42]. An outline of the PID_50_ for inoculation routes suggested low titers for IM, intermediate titers for IN, and high titers for IO in domestic pigs. Regarding IM inoculation titers for assessing the pathogenicity and immunoprotective effect of the ASFV vaccine, the most commonly used dose is 10^2^ or 10^3^ HAD_50_ [21,22,23,31,32,33,34], but there are no guidelines for inoculation doses and routes. These results will contribute to the establishment of a challenge/trial model for the Korean ASFV strain via the IO and IN routes.

The IM challenge has been commonly used in many in vivo ASFV studies, including those assessing the pathogenicity and vaccine candidates’ efficacy/efficiency, because of the highly reproducible clinical disease and easy control of the inoculation dose. However, the IM challenge in evaluating the ASFV vaccine can be incomplete, as pigs inoculated with some ASFV vaccine candidates were protective against IM injection but resistant to IO challenge [43,44]. This implies that alternative challenge routes should be developed to assess the efficacy of ASFV vaccines precisely. Additionally, the IM route, an unnatural route of infection, can cause the virus to enter blood vessels that are closely intertwined within skeletal muscle tissues and directly and quickly contact target cells, leading to the rapid progression of clinical disease [35,45]. As IM inoculation is appropriate for studying the late stage of ASFV infection, it should be avoided when mimicking the natural course of the disease, including research on the pathogenesis mechanism, especially in the early stage of the disease course. DC is one of the most common routes of ASFV infection. However, this requires donor pigs and incurs higher costs owing to longer periods for animal experiments. Moreover, the method cannot easily control the dose quantity and timing. Therefore, the IO and IN routes can be alternative methods for DC. Both routes require appropriate sedation and anesthesia and may not result in a 100% incidence of infection. In this animal experiment, the IO group originally consisted of five pigs; however, one pig was not properly infected despite careful IO inoculation. This might have resulted from the spontaneous swallowing of the inoculum to reduce the time for the virus to adhere to the mucosal surface in the oral cavity. During IN inoculation, sneezing during inoculation may interfere with successful infection. This supports that the IO and IN routes require more careful manipulation to avoid variability in the results.

The route of infection, inoculation dose, and virulence of virus affect the progression and outcome of the clinical manifestation of ASF. Increased inoculation titer of ASFV induces a significant aggressive clinical course with shorter survival period. Immunological and hygienic status of pigs used in the experiment can be one of important determinants of disease progression. SPF pigs presented more severe and rapid clinical course than farm-raised pigs when inoculated with highly virulent strain (Armenia 2008) [46]. With 10^6^ HAD_50_ of highly virulent ASFV-Malawi strain (Malawi-Lil-20/1), IO and IN groups composed of farm-raised pigs had a comparable disease course [35], which was similar to the result of this animal experiment. On the other hands, SPF pigs challenged with 200 and 1000 TCID_50_ of highly virulent ASFV-Chinese strain (SY-18) via the IN route demonstrated more rapid clinical course of infection than the IO route [45]. In this study, 10^3^ times of virus titers for IO or IN route were necessary to induce similar disease progression by IM route. This can be explained by the involvement of mucosa-associated lymphoid tissue (MALT) in the oropharynx or nasopharynx. MALT selectively absorbs virus and may cause immune responses to control virus spread [47]. Presumably, the inoculated virus was partially eliminated in the MALT.

The quantity of live virus excreted from IM-ASFV-infected pigs through oronasal and rectal routes was low, consistent with a previous report [48]. Unless infected blood is exposed to naïve pigs via epistaxis or melena, DC transmission is slow and causes delayed clinical outcomes, making it difficult to select infected pigs at pig farms or contracted wild boar rapidly. Transmission parameters, including basic reproductive ratio (R0) and transmission rate (β), will be shortly assessed with obtained data in this animal experiment to contribute to the establishment of efficient preventive measures. In addition, the response to ASFV infection in individual pigs can vary. In the previous animal experiment with a highly virulent Lithuanian field isolate (LT14/1490), mortality of DC pigs was 94.5%, but one in-contact pig survived without any clinical signs [49]. Low virulent ASFV (MH/P68) induced mild or no clinical signs with transient viremia and seroconversion at four weeks post-exposure [50]. The disease course in pigs can vary depending on the inoculation dose, exposed time, and the immune status of the inoculated pigs.

## 5. Conclusions

This study demonstrated that IO and IN challenges in domestic pigs with 10^6^ HAD_50_ induced a comparable disease course of ASF to that of IM inoculation with 10^3^ HAD_50_. Five DC pigs co-housed with two pigs infected intramuscularly with 10^3^ HAD_50_ showed similar ASF syndrome after 7 days of the latent period. In comparison to DC transmission, one of the most common routes of infection, there were no significant differences between the IO and IN inoculations. However, IM challenge differed from DC in terms of the severity of fever and hemorrhagic lesions in the lymph nodes and spleen. This indicates that the IM route is not appropriate for studying the mechanism of ASFV infection, including the early stage of infection, and that IO and IN challenges with a designated dose can be used as alternatives to IM inoculations in ASFV challenge trials, including assessing the pathogenicity and efficacy of the vaccine.

## Figures and Tables

**Table 1:** Clinical course, viremia, and virus shedding via oral, nasal, and rectal routes in the four groups.

Group	Total Survival Days	Fever	Clinical Signs		Viremia		Virus Shedding					
							Oral Swab		Nasal Swab		Rectal Swab	
		Onset	Onset	Max Scores	Onset	Max Titers ^1^	Onset	Max Titers ^1^	Onset	Max Titers ^1^	Onset	Max Titers ^1^
IO group (*n* = 4)	8.8 ± 1.0	4.5 ± 0.6	6.0 ± 0	17.8 ± 1.0	3.3 ± 0.3	7.7 ± 0.3	4.8 ± 0.3	3.4 ± 0.3	4.3 ± 0.1	5.0 ± 0.1	5.5 ± 1.3	3.2 ± 1.3
IN group (*n* = 5)	8.6 ± 2.3	4.2 ± 0.5	5.0 ± 0.8 ^2^	16.4 ± 2.7 ^2^	3.2 ± 0.4	7.4 ± 0.4	4.4 ± 0.6	2.9 ± 0.6	5.0 ± 0.9	3.7 ± 0.9	7.0 ± 0.5 ^2^	3.8 ± 0.5 ^2^
IM group (*n* = 5)	8.0 ± 1.2	4.6 ± 0.9	5.8 ± 0.5 ^3^	15.5 ± 2.7 ^3^	3.2 ± 0.2	7.3 ± 0.2	4.4 ± 1.3	3.7 ± 1.3	4.8 ± 1.0	4.1 ± 1.0	5.0 ± 0.8 ^3^	4.0 ± 0.8 ^3^
DC group (*n* = 5)	16.6 ± 1.5	11.6 ± 0.9	13.2 ± 0.5	16.4 ± 2.8	9.8 ± 0.1	7.5 ± 0.1	8.2 ± 0.5	3.3 ± 0.5	6.0 ± 0.5	5.0 ± 0.5	11.6 ± 0.5	4.3 ± 0.5
*p*-value ^4^	0.509	0.214	0.043	0.262	0.708	0.258	0.378	0.605	0.393	0.046	0.162	0.549

^1^ Log_10_ genome copies/µL. ^2^ Pig no. 7, which did not show any clinical signs and excrete virus via rectal route, was excluded. ^3^ Pig no. 13, which did not show any clinical signs and excrete virus via rectal route, was excluded. ^4^ The *p*-value was calculated using the Kruskal–Wallis test by ranks after subtracting the 7-day latent period, and onsets of oral and nasal shedding were considered after viremia in the DC group. IO, intraoral; IN, intranasal; IM, intramuscular; DC, direct contact; no., number.

**Table 2:** Summary of clinical signs and gross lesions at necropsy in the IO, IN, IM, and DC groups.

Clinical Signs and Gross Lesions				IO (10^6^)	IN (10^6^)	IM (10^3^)	DC
Clinical sign	fever (>40.0 °C)			4/4	5/5	5/5	5/5
	loss of appetite			4/4	5/5	5/5	5/5
	depression			4/4	5/5	5/5	5/5
	recumbency			4/4	5/5	5/5	5/5
	skin hemorrhage			2/4	1/5	2/5	2/5
	labored breathing and/or cough			0/4	0/5	1/5	5/5
	ocular discharge			0/4	2/5	0/5	0/5
	diarrhea			0/4	0/5	1/5	2/5
	bloody diarrhea			0/4	0/5	0/5	0/5
	joint swelling and lameness			0/4	2/5	0/5	0/5
	nose bleeding			0/4	1/5	0/5	0/5
Gross lesion	lymph nodes	mandibular	enlargement	4/4	5/5	5/5	5/5
			hemorrhage	4/4	1/5	3/5	5/5
		inguinal	enlargement	4/4	5/5	5/5	5/5
			hemorrhage	4/4	2/5	4/5	5/5
		mesenteric	enlargement	4/4	5/5	5/5	5/5
			hemorrhage	4/4	3/5	4/5	5/5
		gastrohepatic	enlargement	4/4	5/5	5/5	5/5
			hemorrhage	4/4	5/5	5/5	5/5
		renal	enlargement	4/4	5/5	5/5	5/5
			hemorrhage	4/4	5/5	5/5	5/5
	kidney		petechiae	4/4	4/5	4/5	5/5
	spleen		thickened	4/4	4/5	2/5	4/5
			dark	4/4	5/5	1/5	4/5
			enlarged	4/4	4/5	2/5	3/5
			friable	4/4	2/5	1/5	3/5
	heart		hydroepicardium	4/4	5/5	5/5	2/5
			epicardial hemorrhage	0/4	4/5	1/5	2/5
	lung		interstitial pneumonia	4/4	5/5	2/5	3/5
			interlobular edema	0/4	2/5	2/5	1/5
	urinary bladder		hemorrhage	0/4	0/5	2/5	0/5
	stomach		petechial hemorrhage	0/4	0/5	1/5	0/5
			hemorrhage	0/4	0/5	0/5	0/5
			ulcer	0/4	0/5	1/5	0/5

IO, intraoral; IN, intranasal; IM, intramuscular; DC, direct contact.

## Data Availability

The data used in this study are available upon request from the corresponding author.
